# Supplementary material for: Influenza A virus reassortment is strain dependent
Source: PLoS Pathog. 2023 Mar 1;19(3):e1011155. doi: 10.1371/journal.ppat.1011155 (PMC10010518; doi:10.1371/journal.ppat.1011155)
Supplement: S1 Text.pdf — (DOCX) [file ppat.1011155.s001.docx]

**SUPPLEMENTARY MATERIALS**

**Influenza A virus reassortment is strain dependent**

Kishana Y. Taylor^1^, Ilechukwu Agu^1^, Ivy José^1^, Sari Mäntynen^1^, A.J. Campbell^1^, Courtney Mattson^1^, Tsui-wen Chou^2^, Bin Zhou^2#^, David Gresham^2^, Elodie Ghedin^2, 3^, Samuel L. Díaz Muñoz^1, 4^

**Figure A.** Correlation between reassortment rate and genetic similarity between strains in each experimental coinfection. Grey shading represents the standard error of the linear model, denoted by the blue line. Points represent each of the 10 experimental coinfections. **A.** Proportion of reassortant progeny generated from each coinfection plotted against the genetic similarity of the parental strains. Genetic similarity calculated using the averaged nucleotide identity among all eight segments. **B.** Proportion of reassortant progeny generated from each coinfection plotted against the genetic similarity of the parental strains. Genetic similarity calculated using the averaged nucleotide identity of all segments excluding antigenic segments HA and NA.

| Strain | CA09 (H1N1) | HK68 (H3N2) | PAN99 (H3N2) | SI86 (H1N1) | TX12 (H3N2) | Segment |
| --- | --- | --- | --- | --- | --- | --- |
| CA09 (H1N1) |  |  |  |  |  | PB2 |
| HK68 (H3N2) | 89.1 |  |  |  |  | PB2 |
| PAN99 (H3N2) | 84.0 | 94.2 |  |  |  | PB2 |
| SI86 (H1N1) | 85.1 | 91.3 | 89.2 |  |  | PB2 |
| TX12 (H3N2) | 83.9 | 92.7 | 97.1 | 88.5 |  | PB2 |
|  |  |  |  |  |  |  |
| Strain | CA09 (H1N1) | HK68 (H3N2) | PAN99 (H3N2) | SI86 (H1N1) | TX12 (H3N2) |  |
| CA09 (H1N1) |  |  |  |  |  | PB1 |
| HK68 (H3N2) | 91.3 |  |  |  |  | PB1 |
| PAN99 (H3N2) | 94.7 | 94.1 |  |  |  | PB1 |
| SI86 (H1N1) | 81.4 | 82.7 | 81.7 |  |  | PB1 |
| TX12 (H3N2) | 93.0 | 92.4 | 96.7 | 81.1 |  | PB1 |
|  |  |  |  |  |  |  |
| Strain | CA09 (H1N1) | HK68 (H3N2) | PAN99 (H3N2) | SI86 (H1N1) | TX12 (H3N2) |  |
| CA09 (H1N1) |  |  |  |  |  | PA |
| HK68 (H3N2) | 82.9 |  |  |  |  | PA |
| PAN99 (H3N2) | 83.3 | 93.4 |  |  |  | PA |
| SI86 (H1N1) | 83.1 | 94.4 | 91.3 |  |  | PA |
| TX12 (H3N2) | 83.3 | 92.2 | 97.0 | 90.3 |  | PA |
|  |  |  |  |  |  |  |
| Strain | CA09 (H1N1) | HK68 (H3N2) | PAN99 (H3N2) | SI86 (H1N1) | TX12 (H3N2) |  |
| CA09 (H1N1) |  |  |  |  |  | HA |
| HK68 (H3N2) | 53.2 |  |  |  |  | HA |
| PAN99 (H3N2) | 54.4 | 89.2 |  |  |  | HA |
| SI86 (H1N1) | 77.3 | 53.8 | 54.9 |  |  | HA |
| TX12 (H3N2) | 53.9 | 85.9 | 94.4 | 66.9 |  | HA |
|  |  |  |  |  |  |  |
| Strain | CA09 (H1N1) | HK68 (H3N2) | PAN99 (H3N2) | SI86 (H1N1) | TX12 (H3N2) |  |
| CA09 (H1N1) |  |  |  |  |  | NP |
| HK68 (H3N2) | 84.7 |  |  |  |  | NP |
| PAN99 (H3N2) | 82.9 | 92.9 |  |  |  | NP |
| SI86 (H1N1) | 84.6 | 94.2 | 90.3 |  |  | NP |
| TX12 (H3N2) | 82.2 | 91.6 | 96.7 | 89.1 |  | NP |
|  |  |  |  |  |  |  |
| Strain | CA09 (H1N1) | HK68 (H3N2) | PAN99 (H3N2) | SI86 (H1N1) | TX12 (H3N2) |  |
| CA09 (H1N1) |  |  |  |  |  | NA |
| HK68 (H3N2) | 52.0 |  |  |  |  | NA |
| PAN99 (H3N2) | 53.3 | 90.2 |  |  |  | NA |
| SI86 (H1N1) | 79.9 | 50.7 | 52.4 |  |  | NA |
| TX12 (H3N2) | 52.2 | 87.9 | 95.1 | 51.1 |  | NA |
|  |  |  |  |  |  |  |
| Strain | CA09 (H1N1) | HK68 (H3N2) | PAN99 (H3N2) | SI86 (H1N1) | TX12 (H3N2) |  |
| CA09 (H1N1) |  |  |  |  |  | M |
| HK68 (H3N2) | 88.8 |  |  |  |  | M |
| PAN99 (H3N2) | 86.9 | 96.3 |  |  |  | M |
| SI86 (H1N1) | 88.1 | 95.7 | 93.6 |  |  | M |
| TX12 (H3N2) | 86.7 | 94.6 | 97.3 | 92.4 |  | M |
|  |  |  |  |  |  |  |
| Strain | CA09 (H1N1) | HK68 (H3N2) | PAN99 (H3N2) | SI86 (H1N1) | TX12 (H3N2) |  |
| CA09 (H1N1) |  |  |  |  |  | NS |
| HK68 (H3N2) | 82.5 |  |  |  |  | NS |
| PAN99 (H3N2) | 81.5 | 94.2 |  |  |  | NS |
| SI86 (H1N1) | 82.7 | 94.2 | 89.5 |  |  | NS |
| TX12 (H3N2) | 81.2 | 93.0 | 97.1 | 88.8 |  | NS |

**Table A.** Pairwise nucleotide identity between strains used in experimental coinfections by segment.

| Strains in Coinfection | Total Segments | Mean Proportion | Std Dev Proportion | Number of Segments That Assort Randomly |
| --- | --- | --- | --- | --- |
| HK68(H3N2)_TX12(H3N2) | 8 | 0.967 | 0.0289 | 0 |
| CA09(H1N1)_PAN99(H3N2) | 8 | 0.888 | 0.147 | 1 |
| CA09(H1N1)_TX12(H3N2) | 8 | 0.708 | 0.138 | 2 |
| PAN99(H3N2)_SI86(H1N1) | 8 | 0.615 | 0.0839 | 6 |
| CA09(H1N1)_SI86(H1N1) | 8 | 0.727 | 0.118 | 1 |
| SI86(H1N1)_TX12(H3N2) | 8 | 0.825 | 0.131 | 1 |
| CA09(H1N1)_HK68(H3N2) | 8 | 0.993 | 0.0115 | 0 |
| HK68(H3N2)_PAN99(H3N2) | 8 | 0.757 | 0.131 | 2 |
| PAN99(H3N2)_TX12(H3N2) | 8 | 0.968 | 0.0221 | 0 |
| HK68(H3N2)_SI86(H3N2) | 8 | 0.895 | 0.0778 | 0 |

**Table B.** Representation of segments among progeny plaque isolates for each experimental coinfection. Each genotype entered the coinfection at a 0.5 proportion. For each coinfection, across all segments, the mean proportion of each strain genotype is shown. The number of segments that show random assortment (i.e. fall within the confidence interval for a 50:50 ratio) are listed in the last column.

**Figure B.** Relationship between the reassortment rate and the number of segments that individually assort randomly among the progeny.

**Figure C.** Segment representation in progeny plaque isolates with regard to strain, using only reassortant plaques. **A.** Plot shows frequency of each strain’s allele for each segment in plaque isolates. **B.** Depicts which segment frequencies are within (blue points) or outside (red points) the confidence interval for 50:50 distribution of strain alleles among plaque isolates.

**Detailed Linked Multilocus Genotype by Sequencing** (LMGSeq) **Methods**

*LMGSeq Conceptual overview*

LMGSeq uses a nested barcode design to enable genotyping of many loci in thousands of individuals while retaining genetic linkage (Figure D). In our design, the forward primer contains an 8bp barcode that represents a 96-well plate and the reverse primer contains another 8bp barcode that represents each well position in a 96-well plate; respectively these are a cross barcode (representing each experimental coinfection) and a sample barcode (representing each progeny plaque isolate, i.e. picked plaques). Thus, each read can be traced to a specific experimental coinfection and sample. Multiple loci are targeted with different gene specific regions (see Table C). The barcodes are flanked by the gene specific region and a portion of the Illumina sequencing primer. In the first PCR reaction (PCR1), each well gets a cocktail of 10 forward, gene-specific primers barcoded for that plate (cross) and a single reverse primer (which binds to the uni13 region, shared among all segments) barcoded for that well (sample). Because all amplicons from PCR1 are barcoded and share flanking regions, a second PCR (PCR2) on all amplicons is conducted to add the remainder of the sequencing primers and the adaptors for Illumina sequencing. Thus, library construction was PCR-based and the final pooled library was purified before paired end sequencing (PE300) on the Illumina MiSeq. Note that because the barcodes are in a custom position, the index reads and automatic demultiplexing on the Illumina MiSeq should be turned off to prevent failure of the run (custom demultiplexing is described below).

To provide an idea of the throughput of the LMGSeq approach, in this paper, the Illumina MiSeq run yielded 25.7 M reads passing filter of which 7.5% were the PhiX control library. Within this run we included a total of 20 experimental coinfections and isolated 96 plaques for each. On average, read depth for each of 10 loci (in each cross and each sample) was 1,293x, allowing robust determination of the genotype.

**Figure D.** LMGSeq amplicon design and library construction.

Complete sequences of all primers are provided in primer order form spreadsheets, included in the GitHub repository (forward primers: influenza_gbs6_mix_c_segment_spp_primers.xls; reverse primers: influenza_gbs6_primers_uni13_96_plate.xls). PCR2 primers were:

Forward: 5’-AATGATACGGCGACCACCGAGATCTACACTCTTTCCCTACACGAC-3'

Reverse: 5'-CAAGCAGAAGACGGCATACGAGATGTGACTGGAGTTCAGACGTG-3'

The design of primers used in sequencing reactions is discussed in the sections below.

*Gene specific amplicon regions*

We designed amplicons for each of the eight segments: for antigenic proteins, which show high sequence variability we designed two primers, one for each human influenza virus subtype for a total of 10 loci. The amplicons were generated by priming to the uni13 at the 5’ end of influenza vRNAs, which is shared among all influenza A strains [1] and to an internal region specific to each segment (Table C).

| Genome Segment | Locus Name | Forward Gene Specific Region | Reverse IAV-universal Region* |
| --- | --- | --- | --- |
| PB2 | PB2f | GGCAATTCTCC | AGTAGAAACAAGG |
| PB1 | PB1c | GCACATGGTCC | AGTAGAAACAAGG |
| PA | PAc | GCTCTTAGGGACAA | AGTAGAAACAAGG |
| HA *(H1 specific)* | HA1b | AGTTGATGATGG | AGTAGAAACAAGG |
| HA *(H3 specific)* | HA3a | GGATTTCCTTTGCCATATCA | AGTAGAAACAAGG |
| NP | NPd | ATGGCAGCATTC | AGTAGAAACAAGG |
| NA *(N1 specific)* | NA1c | AATGGTGTTTGGATAGG | AGTAGAAACAAGG |
| NA *(N2 specific)* | NA3a | GGTGCTTTTATGTGGAGTTG | AGTAGAAACAAGG |
| M | Mg | CATGAGAACAGAATGGT | AGTAGAAACAAGG |
| NS | NS1d | GAGGATGTCAAAAATGC | AGTAGAAACAAGG |

**Table C.** Gene specific regions of primers used in this study. *All reverse primers targeted the uni13 region, which is shared among all influenza A genome segments.

*Barcode design*

Barcodes were selected using the approach of Buschmann and Bystrykh [2,3] as implemented in the R package DNABarcodes. Eight base pair barcodes were generated to maximize the Levenshtein distances (d = 3, Sequence-Levenshtein in paper), to guarantee correction of at least one error (substitution, insertion, deletion). Because the Illumina sequencing platform does not have trouble with triplet homopolymers, these were allowed in the barcodes. However, barcodes containing the GGC motif, which is known to be associated with sequencing errors in this platform, were not selected.

*Demultiplexing and read merging*

Starting from the raw FASTQ files, each read was assigned to a specific sample (plaque isolate) within a specific cross (experimental coinfection). First, cutadapt v2.6 [4] was used to identify the sample and cross barcode simultaneously, using the linked adaptor option in paired end mode, allowing exact matches only to a full 8bp barcode sequence. This approach together with the amplicon design allowed several checks on the barcodes. First, the first bases sequenced after the sequencing primer on Illumina platforms are the most accurate, thus we positioned our barcodes as the very first 8bps to be sequenced to allow precise determination of the sample (on read 1, R1) and cross barcode (on read 2, R2). The sequencing file was first demultiplexed (without trimming) according to these first barcodes on R1 and R2 to split into individual files for each individual sample (plaque isolate).

cutadapt -g file:barcodes5.fasta -G file:barcodes3rev.fasta -j 0 -O 8 --no-indels --action=none --discard-untrimmed -o trimmed_{name1}_{name2}_$1_R1.fastq -p trimmed_{name1}_{name2}_$1_R2.fastq data/$1_R1.fastq data/$1_R2.fastq

Second, starting from the demultiplexed files, we further quality controlled the reads using the reverse complement of barcodes to verify both the sample and cross barcode on the paired read. Thus, only reads that had complete and perfectly matching sequences independently on both reads were trimmed and selected for further analysis. Using this approach we trimmed barcodes from each sample file, corresponding to a single plaque isolate.

cutadapt -a ${barcode5}...${barcode3} -A ${barcode3rev}...${barcode5rev} -O 8 -o ${cross}_${sample}_R1.fastq -p ${cross}_${sample}_R2.fastq trimmed_${cross}_${sample}_$1_R1.fastq trimmed_${cross}_${sample}_$1_R2.fastq

We then proceeded to remove the regions of our primers that targeted the influenza genome. First, we trimmed the uni13 sequence (see Table C), which was used as the reverse primer targeting this conserved region at the end of all influenza A genome segments. Matches did not have to be exact, but the matches were required on R1 and R2.

cutadapt -a "CCTTGTTTCTACT" -G "^AGTAGAAACAAGG" -o uni13_${prefix}_R1.fastq -p uni13_${prefix}_R2.fastq ${prefix}_R1.fastq ${prefix}_R2.fastq

Second, the segment specific region of the forward primer was used to generate individual files for each locus in each sample and trim the sequences. The command allowed no errors in the bases matched, but the length of the match did not have to be exact, only had to be a minimum of 10bp (gene specific regions varied from 11-20bp).

cutadapt -g file:shared/segment_specific_primers.fasta -A file: shared/segment_specific_primers_rev.fasta -e 0.0 -O 10 --no-indels -o ${cross_sample}_{name}_R1.fastq -p ${cross_sample}_{name}_R2.fastq ${prefix}_R1.fastq ${prefix}_R2.fastq > cutadapt_${cross_sample}_primer_demultiplexing.txt

The end result of this demultiplexing script is a collection of 10 locus-specific FASTQ files for each sample in each cross.

*Amplicon Curation and Strain assignment*

The paired reads were merged into a single read using PEAR v0.9.6 [5].

pear -f ${prefix}_R1.fastq -r ${prefix}_R2.fastq -o ${cross}/${prefix}_merged.fastq -v 3

A database containing all possible amplicon sequences from all strains used in the experiment, was subset to include only the strains included in each cross (experimental coinfection). This database customized to each cross was used as a reference in a usearch v8.1.1861 [6] command that yielded the best global match for each read at a ≥98 percent identity (PID) threshold.

usearch -usearch_global ${file1} -db ${cross}/${cross}_reference_database.fasta -id 0.98 -blast6out usearch/all/${prefix}_98_merged.b6 -strand both

The resulting BLAST output format files were screened for database matches that did not correspond to the locus of the analyzed file; these were cross-amplifications of primers with non-target genome segments. For each locus in each sample, the script tallied the number of strain matches and lists the number of matches to the strain with most matches and the total number of matches (assigning a genotype to each locus was a binary choice between one of the coinfecting parental strains). These files (one per cross) were then used for downstream analyses in R v3.6.3 [7], which assigned genotypes and analyzed the data.

*Genotype Assignment and Analyses*

The output files were imported recursively in R to generate a data frame that had cross/sample/locus combination as a row. We had two loci for each antigenic segment (NA and HA) targeting each influenza subtype (H1N1 and H3N2). When we (unexpectedly) had samples where the antigenic loci cross-amplified between subtypes, we further processed the data to assign a subtype to each sample using quantity and quality thresholds that we established using control samples that were individual strains subjected to LMGSeq. Altering these criteria did not qualitatively affect the results.

The endpoint of the LMGSeq was genotype calls for each segment in each sample (plaque isolate). These data were the starting point for the analyses described in the manuscript and full details on how the figures and results were generated are provided in annotated code in the Github repository: [https://github.com/sociovirology/human_influenza_LMGSeq](https://github.com/sociovirology/human_influenza_GbBSeq).

**References for Supplementary Materials**

1. Zhou B, Donnelly ME, Scholes DT, George KS, Hatta M, Kawaoka Y, et al. Single-reaction genomic amplification accelerates sequencing and vaccine production for classical and Swine origin human influenza a viruses. J Virol. 2009;83: 10309–10313. doi:10.1128/jvi.01109-09

2. Buschmann T, Zhang R, Brash DE, Bystrykh LV. Enhancing the detection of barcoded reads in high throughput DNA sequencing data by controlling the false discovery rate. Bmc Bioinformatics. 2014;15: 264. doi:10.1186/1471-2105-15-264

3. Buschmann T, Bystrykh LV. Levenshtein error-correcting barcodes for multiplexed DNA sequencing. Bmc Bioinformatics. 2013;14: 272. doi:10.1186/1471-2105-14-272

4. Martin M. Cutadapt removes adapter sequences from high-throughput sequencing reads. Embnet J. 2011;17: 10–12. doi:10.14806/ej.17.1.200

5. Zhang J, Kobert K, Flouri T, Stamatakis A. PEAR: a fast and accurate Illumina Paired-End reAd mergeR. Bioinformatics. 2014;30: 614–620. doi:10.1093/bioinformatics/btt593

6. Edgar RC. Search and clustering orders of magnitude faster than BLAST. Bioinformatics. 2010;26: 2460–2461. doi:10.1093/bioinformatics/btq461

7. Team RC. R: A Language and Environment for Statistical Computing. 2020. Available: <https://www.R-project.org/>
