# Supplementary material for: Influenza A virus reassortment is strain dependent
Source: PLoS Pathog. 2023 Mar 1;19(3):e1011155. doi: 10.1371/journal.ppat.1011155 (PMC10010518; doi:10.1371/journal.ppat.1011155)
Supplement: Attachment — Submitted filename: response_to_reviewers_v3.docx [file ppat.1011155.s002.docx]

Dear Jacob S. Yount, Ana Fernandez-Sesma, Kasturi Haldar, and Michael Malim:

We appreciate the reviews of our manuscript (PPATHOGENS-D-22-01910). We are writing to provide a response to the reviews and to summarize the changes

Please see below our point-by-point response to reviewer comments and the changes we have made in response to each comment.

We have also included two versions of the manuscript with our resubmission, per your guidelines, one with highlighted changes and a clean version

We appreciate your time and look forward to your response.

Warm regards,

Sam Díaz-Muñoz on behalf of co-authors

**Response to Reviewers**

**We would first like to thank the reviewers for the reviews and especially for the positive comments on the manuscript. We very much appreciate the constructive comments as they will go a long way to improve our manuscript. Please see below our response to each comment.**

**Part I - Summary**
Please use this section to discuss strengths/weaknesses of study, novelty/significance, general execution and scholarship.

Reviewer #1: In their manuscript, Taylor et. al. describe their study of reassortment of human seasonal influenza strains using in vitro and in silico approaches. Overall, the paper is well written, relatively clear, and the data is robust and novel. The findings are of great interest, especially in regard to pandemic prevention and future events which may result in novel, reassortant strains.

**We thank Reviewer #1 for these positive comments. We are very happy that the relevance of our study was clear and that our data can contribute to move the field forward while informing public health.**

However, some issues should be addressed before the paper is acceptable for publication.

1) The authors should spend a bit more time in the introduction discussing the overall risk of coinfection with both a A/H1N1 and A/H3N2 strain within a given season to increase public health relevance.

**This is a great point. We have included a few sentences to discuss the following points: a) circulation of human subtypes in swine; b) co-circulation and reassortment of human a A/H1N1 and A/H3N2 within seasons; and c) the emergence and circulation of H1N2, which highlight the risks of coinfection in a given season.**

2) How do the strains utilized in this study differ from their egg passaged original stocks? Was sequencing performed both prior and post initial passaging in MDCK? How many passages were performed in MDCK before use in their experiments? Was each strain ensured to be of the same passage and same MDCK-associated adaptive mutations before coinfection experimentation began?

**Part of our rationale for strain selection was viruses that did not have severely skewed adaptation to MDCK cells and that could plaque efficiently in these cells. Our strains did not have major differences in replication or plaquing in MDCK cells. We created stocks in MDCK cells from the vials we received from Dr. Ted Ross (egg passaged). Our stocks were created by amplifying once in MDCK cells. All strains were subjected to the same number of passages, which we now have noted in the methods section. The differences in titers from the original egg passaged stocks (2.9 x 10^6^ - 5.25 x 10^8^) were much narrower at the end of our passaging in the lab, which yielded a much tighter range (1.66x10^7^ - 1.83x10^8^ pfu/mL) of titers in MDCK cells. We conducted sequencing of our stocks (post-passaging) in the regions we used for genotyping (ca ~18% of the genome), but did not search for MDCK-associated mutations as we didn’t note differences in MDCK adaptation, as outlined above. Most importantly, the slight differences in titers do not map to any of the reassortment outcomes.**

3) How was coinfection of single cells detected and confirmed? It would be ideal to show that the MOI utilized resulted in a significant (and equivalent) number of cells was coinfected in each experimental condition.

**We did not directly examine coinfection of single cells. However, reassortants can only emerge from coinfected cells and we showed that increasing the MOI causes an exponential increase in reasortants (which would not include all coinfected cells) in the HK68 and CA09 pairing. Therefore, we feel confident that the MOI we chose was causing increased coinfection.**

**We think it is important to understand the overall pattern of a simultaneous inoculation with two strains and not limit it only to coinfected cells as we think the fact that some cells are not coinfected or are coinfected and do not generate reassortants is important. Here our main goal was to conduct classical experimental coinfections of intact strains, to gain a sense of the range of potential reassortment of simultaneous infection at a population level. Follow-up work in our lab will examine potential strain-specific patterns in infection and coinfection and how this changes according to the MOI for each strain and strain combination. We will also pursue single cell approaches (e.g. scRNAseq) in the future for select experiments as these are much lower throughput approaches.**

4) It is highly unclear if infections were performed first them plaque assays were used to isolate individual, viable viral strains or if mock infections were performed and individually infected cells were chosen. The former experiment represents true coinfection and production of single, viable strains while the second just represents mixture within a cell and could result in defective viral particles clinging to the surface of the cell. Please consider describing the experimentation in detail to make clear.

**We thank the reviewer for highlighting that this extremely important point was not clear. The answer is that “infections were performed first then plaque assays were used to isolate individual, viable viral strains”. Thus, as the reviewer states, our experiment “represents true coinfection and production of single, viable strains”. This was a very important element of our study design that we wanted to get right. We have added the wording specified by the reviewer and a figure (now Figure 1) to communicate our study design more quickly and neatly.**

5) 16.3x seems a bit low for coverage of such an important segment as PB1 – what optimization was performed to try to increase this yield, especially for a strain that has important implications for viral replication. This does not appear to be dealt with experimentally in the protocol and just in silico, which is likely insufficient for overall study design.

**We appreciate the observation on coverage. This would indeed be low coverage if one’s goal was to determine an unknown sequence. However, our goal in using sequencing was to determine if a reassortment event had occurred. That is, we needed enough information to make a binary call on which parental segment was represented in each plaque.**

**At the conception of our experiment, we were aware that there would be differences in coverage, particularly in the polymerase segments, as we note in the introduction. This is why we designed the LMGSeq approach to rely on sequencing: because the strains we selected are distinguished by multiple single nucleotide variants (SNVs) (as opposed to other reassortment detection approaches which rely on a single SNV). Thus, in theory (i.e. assuming clonality of the plaque) we only need one read to determine the genotype. Even if the very low error rate of Illumina sequencing were to lead to an error in one of the segment’s informative bases, we have multiple other SNVs to assign each read. In practice we have more than one read and each read increases the statistical confidence in the call. By examining our empirical data, we can see that even for PB1, our assay worked as designed: 92% of PB1 segments were assigned to a strain by >80% of reads, which is a higher rate than the 88.59% of segments overall that were assigned with >80% of reads. Thus, we have high confidence in our PB1 segment assignments and our assay design for LMGSeq worked as intended.**

**In short, although the coverage for PB1 was indeed lower than for other segments, it is more than sufficient to provide robust statistical confidence to assign a genotype and thus infer reassortment, the goal of this study.**

6) A panel of 5 viruses (3x H3N2 and 2x H1N1) from 2 geographic locations (Asia and North America) does not really constitute as wide of a panel of viruses as implicated in the text, please consider toning down the language and further classifying the full differences between these strains that make them the appropriate choice for these experiments, especially since very few of them would have been cocirculating at any given time. More biologically relevant, currently circulating strains (such as those from before and after COVID-19) may be a better representation of the true risks of this reassortment at the current time.

**We were trying to emphasize the novelty of using multiple strains in our experimental coinfections. We have toned down the language in the manuscript. We deliberately chose strains that were not co-circulating as our suspicion was that co-circulating strains (particularly from the same subtype) would have higher reassortment. We derive this suspicion from ample phylogenetic evidence showing that intrasubtype reassortment occurs within seasons (references in updated introduction). These data are relevant as it is more difficult to detect reassortment phylogenetically from epidemiological surveillance data because a) sequence divergence is required to detect a reassortment event and b) the reassortant has to be successful enough to emerge and circulate. Moreover, as we note in the updated text (responding to point 1 above)** **reassortment of H3N2 strains with co-circulating 2009 pandemic H1N1 *and* pre-pandemic H1N1 strains has been detected phylogenetically (Gregory et al., 2002; Mukherjee et al., 2012) and some of these H1N2 strains circulated widely and even became the dominant strain in some locations (Mukherjee et al., 2012).**

**Although we agree that a current assessment of pre- and post-COVID-19 pandemic strains would be a better representation of risks at the current time, such a study is beyond the scope of the current study. The goal of our manuscript was not to conduct a public health risk assessment, but rather to determine the factors that contributed to reassortment. By showing that specific strains can increase or decrease reassortment frequency, we believe we are providing a guideline for experiments that can be conducted on actively circulating strains to provide public health assessments of reassortment risk. Such studies are precisely the type of follow-up study we are planning as we move into a much-changed respiratory virus landscape with the “triple demic” including co-circulating A/H1N1, A/H3N2 and the present influenza swine reservoir. We are excited for our group to conduct risk assessment studies and to provide the tools for other groups and public health agencies to do the same.**

**References:**

**Mukherjee TR, Agrawal AS, Chakrabarti S, Chawla-Sarkar M. 2012. Full genomic analysis of an influenza A (H1N2) virus identified during 2009 pandemic in Eastern India: evidence of reassortment event between co-circulating A(H1N1)pdm09 and A/Brisbane/10/2007-like H3N2 strains. Virol J 9:233. doi:10.1186/1743-422x-9-233**

**Gregory V, Bennett M, Orkhan MH, Hajjar SA, Varsano N, Mendelson E, Zambon M, Ellis J, Hay A, Lin YP. 2002. Emergence of Influenza A H1N2 Reassortant Viruses in the Human Population during 2001. Virology 300:1–7. doi:10.1006/viro.2002.1513**

7) Figure 2, 6A, is extremely hard to interpret in grayscale…please consider making colors a bit more definitive.

**We thank the reviewer for pointing this out and have increased contrast by 35% in this panel.**

8) As mentioned in the results and discussion, the authors mention some issues with undetectable results or insignificance due to sample size constraints. While the calculation how many plaques would be required is interesting (and justifiable for some analyses), the lack of additional data does diminish the final result of the paper and further individual subtype recombination may need to be explored.

**We agree that individual subtype reassortment and our future work is aimed in that direction. Part of the novelty of our work is precisely that our data enabled statistical tests at all. While highlighting that advance in the literature, we also wanted to acknowledge the limitations and where we have to go as a field to answer these questions from a robust statistical perspective.**

9) The discussion about highly productive infection in the discussion may not be justified as the authors do not directly measure virulence or any sort of viral replication as associated with each individual plaque isolate.

**Thank you for bringing up this point as it provides the opportunity to clarify what we meant when we said – “highly productive infection”. That should read highly productive *co-infection*. In this paper we wanted to focus strictly on the outcomes of co-infection, not on the characteristics and fitness of reassortants, which of course is an important question, albeit one that is conflated with reassortment itself.**

10) Do the authors intend to perform these studies using potentially zoonotic strains as well as indicated in the last paragraph of the discussion? This would be extremely biologically relevant, especially with the high frequency of A/H5Nx 2.3.4.4b infections being detected in mammals in Europe and North America as well as the large influenza season currently affecting the northern hemisphere.

**These experiments would indeed be extremely biologically relevant but would be challenging to conduct for our laboratory from a biosafety and regulatory perspective. However, our intention is to pursue collaborations that would enable experimentation on high-pathogenic and potentially zoonotic strains and also to make our approach available to those labs that have the ability to conduct these experiments.**

11) Was any attempt made to perform these analyses on clinical isolates from true coinfections to correlate the number of reassortants observed with what is observed in vitro/in silico?

**We do not have access to clinical samples that are from true coinfections – these data are seldom collected and require collaborations at the point of care to identify true coinfection. However, we are pursuing collaborations with clinicians to address this very point. Please also refer to our response to point 6 above.**

Reviewer #2: Taylor et al. establish a genotyping approach and quantify segmental reassortment between five seasonal influenza viruses of H1N1 and H3N2 subtypes collected over 41 years, and identify several interesting details on reassortment. These include (a) some viruses can influence reassortment independent of its co-infecting partner (b) reassortment involves all eight segments with little preference towards specific segments, and (c) linkages between segments were found, but they were not related to sequence similarity or subtype. Overall, this is a nice study that significantly advances our understanding of segmental reassortment. The Results and Figures were presented clearly, but the text could be clearer.

**We thank Reviewer #2 for the kind words about our study.**

**Part II – Major Issues: Key Experiments Required for Acceptance**
Please use this section to detail the key new experiments or modifications of existing experiments that should be absolutely required to validate study conclusions.

Generally, there should be no more than 3 such required experiments or major modifications for a "Major Revision" recommendation. If more than 3 experiments are necessary to validate the study conclusions, then you are encouraged to recommend "Reject".

Reviewer #1: Please see Part I above

**We refer to our response to Reviewer #1, in Part I above. In summary, we have either made modifications or present data for all the points raised by reviewer #1.**

Reviewer #2: 1. Too much hype is made that the viruses span 41 years, "a diverse panel of human influenza virus strains to encompass 41 years of epidemics, multiple geographic locations, and both circulating human subtypes A/H1N1 and A/H3N2". However, this phrase has some issues.

**We agree with Reviewer #2 and appreciate the opportunity to more precisely convey the points we were trying to make and have modified the phrase as indicated below in response to the reviewer’s comments. The new phrase throughout the manuscript now reads:**

**“a diverse panel of human influenza virus strains encompassing two pandemics (swine and avian origin); three specific epidemics; and both circulating human subtypes A/H1N1 and A/H3N2.”**

- The five strains do not encompass epidemics for 41 years. Instead, they represent two pandemics and some other specific epidemics. CA09 originated from swine, and the origin of HK68 is assumed to be birds.

**We have adjusted our wording to reflect the important information Reviewer #2 has conveyed.**

- The mention of geography is jarring because human influenza is a globally circulating virus.

**We have removed the mention of geography.**

- I can accept that the strains represent a diverse panel of human influenza 'A' viruses.

**We are glad you agree with it being a diverse panel; this is the main point we were trying to make and appreciate the opportunity to correct our language if our wording was leaning towards hype.**

2. Interestingly, all three strains that tended to increase reassortment were older (PAN99, HK68, and SI86), which likely underwent more passage than recent viruses. Can adaptation to cells influence these findings?

**We thank the reviewer for this observation. Adaptation to cells could certainly influence our findings. This was part of our rationale for strain selection, we wanted strains that did not have severely skewed adaptation and that were able to plaque efficiently. We created stocks in MDCK cells from the vials we received from Dr. Ted Ross and the titers were within a relatively tight range (1.66x10^7^** - **1.83x10^8^ pfu/mL). Most importantly, these differences in titers do not map to reassortment outcomes; also, as a point of clarification, we note that HK68 coinfections yielded the *lowest* average reassortment frequencies.**

3. Results "Thus, reassortment frequencies are presented using all data, which is a conservative approach because inclusion of genotypes that are missing segments decreases the chance of detecting a reassortant."
Please clarify whether incomplete genomes were included/excluded.

**We have further clarified that incomplete genomes were included. The sentence now reads:**

**“Thus, reassortment frequencies are presented using all data (i.e. including incomplete genotypes), which is a conservative approach because inclusion of genotypes that are missing segments decreases the chance of detecting a reassortant.”**

4. Parts of the Discussion that could be easier to read.

**We appreciate these corrections.**

Third, .... with homologous linkages preferred"

**The sentence now reads:**

**“Third, we found evidence of non-random pairwise segment associations. Specifically, most strain combinations yielded homologous linkages but this outcome again was not related to strain similarity or shared subtype.”**

Fourth, "we found a range" in the number of unique genotypes generated by each coinfection

**The sentence now reads:**

**“Fourth, the number of unique genotypes generated by each coinfection spanned a wide range (1-36), again suggesting emergent outcomes from distinct strain combinations.”**

"An important study design and conceptual element regarding this work bears discussion"

**The sentence now reads:**

**“Our study design has implications for data analysis and interpretation of all studies examining reassortment: we sought to examine the outcomes of coinfection of intact strains and any reassortment potential.”**

**Part III – Minor Issues: Editorial and Data Presentation Modifications**
Please use this section for editorial suggestions as well as relatively minor modifications of existing data that would enhance clarity.

Reviewer #1: Please see Part I above

**We refer to our response to Reviewer #1, in Part I above.**

Reviewer #2: Replace "Isolation" with Region/Country in Table 1.

**We thank the reviewer for this suggestion and have modified the heading in Table 1.**

"protocol was independently developed and varies in some experimental steps". Can the author briefly mention these steps in the subsequent lines?

**We thank the reviewer for this suggestion and have included more detail in how our method varies from the referenced studies that use similar methodology in other fields.**
